# Changes in Blood Pressure and Lipid Levels in Young Women Consuming a Vitamin D-Fortified Skimmed Milk: A Randomised Controlled Trial

**DOI:** 10.3390/nu5124966

**Published:** 2013-12-05

**Authors:** Laura Toxqui, Ruth Blanco-Rojo, Ione Wright, Ana M. Pérez-Granados, M. Pilar Vaquero

**Affiliations:** Department of Metabolism and Nutrition, Institute of Food Science, Technology and Nutrition (ICTAN), Spanish National Research Council (CSIC), C/José Antonio Novais 10, 28040 Madrid, Spain; E-Mails: laura.toxqui@ictan.csic.es (L.T.); ruth.blanco@ictan.csic.es (R.B.-R.); ione.wright@ictan.csic.es (I.W.); granados@ictan.csic.es (A.M.P.-G.)

**Keywords:** vitamin D, food fortification, blood pressure, cholesterol, randomised controlled trial, women, dairy product

## Abstract

Vitamin D exerts a variety of extra-skeletal functions. Aim: to know the effects of the consumption of a vitamin D-fortified skimmed milk on glucose, lipid profile, and blood pressure in young women. Methods: a randomised, placebo-controlled, double-blind parallel-group trial of 16 weeks duration was conducted in young women with low iron stores who consumed a skimmed milk fortified with iron and 200 IU/day (5 μg) of vitamin D (D-fortified group, *n* = 55), or a placebo without vitamin D (D-placebo group, *n* = 54). A reference group (*n* = 56) of iron-sufficient women was also recruited. Results: baseline serum 25-hydroxyvitamin D was inversely correlated with total-cholesterol (*r* = −0.176, *p* = 0.023) and low density lipoprotein-cholesterol (LDL-chol) (*r* = −0.176, *p* = 0.024). During the assay, LDL-cholesterol increased in the D-placebo group (*p* = 0.005) while it tended to decrease in the D-fortified group (*p* = 0.07). Neither group displayed changes in total-cholesterol, high density lipoprotein-cholesterol (HDL-chol), triglycerides or glucose levels. Systolic (*p* = 0.017) and diastolic (*p* = 0.010) blood pressure decreased during the assay in the D-fortified group without significant differences compared to the D-placebo. Conclusion: consumption of a dairy product fortified with vitamin D reduces systolic and diastolic blood pressure but does not change lipid levels in young women.

## 1. Introduction

It is well known that vitamin D is essential for calcium absorption and bone health. Moreover, a number of extra-skeletal effects have been attributed to this vitamin since the vitamin D receptor and the enzyme 1α-hydroxylase, which converts 25-hydroxyvitamin D (25OHD) to its active form 1,25-dihydroxyvitamin D, are present in most body tissues [1,2]. Therefore, research on the role of vitamin D in chronic illnesses including cancer, diabetes, and cardiovascular and autoimmune diseases is an emerging field [3].

Low vitamin D status, assessed by 25OHD levels, has been associated with increased risk of major cardiovascular events, cardiovascular mortality, and all-cause mortality [4,5,6]. However, the associations observed with specific cardiovascular risk markers are not consistent or are contradictory [1,7,8]. Vitamin D may influence cardiovascular disease risk by regulating blood pressure through the rennin-angiotensin system [9], by modulating serum lipids levels [1], or by stimulating insulin secretion [10]. Moreover, reports on the cardiovascular effects of vitamin D in healthy people, including young women, are scarce. However, randomised clinical trials are scarce and further research is needed to clarify this relationship [1,7,11].

Recently, a relationship between vitamin D and iron deficiencies has been suggested [12]. Intervention studies in menstruating women show that iron plays a role in bone remodelling and that vitamin D enhances erythropoiesis [13,14]. Therefore, the metabolic pathways of both micronutrients may be interrelated.

There is variability in the Recommended Dietary Allowances (RDA) for different countries. The Spanish RDA for adults are established at 200 IU (5 μg) per day [15,16] while the United States and Canada RDA are set at 600 IU (15 µg) per day [17]. In spite of being a sunny Mediterranean country, Spain presents high prevalence of vitamin D deficiency [18]. Therefore, food fortification can be useful to prevent vitamin D deficiency, achieve peak bone mass and maintain bone health [19]. There are different fortification practices in the world; countries such as Canada and the United States of America have developed mandatory fortification in several staple food items, however fortification with vitamin D is optional in the European countries [20].

The aim of the present study was to know: (1) whether there is an association between vitamin D status and lipid levels; and (2) whether consumption of a vitamin D-fortified dairy product, compared to a placebo, modified the lipid profile, blood pressure, or glucose levels in young women.

## 2. Experimental Section

### 2.1. Subjects

Healthy, 18–35 years old, non-smoking, non-pregnant, and non-breast-feeding Caucasian women were recruited. Those with low iron stores (serum ferritin ≤ 30 ng/mL, haemoglobin ≥ 11 g/dL) were assigned to one of the nutritional intervention groups, while those with normal iron stores (serum ferritin > 30 ng/mL, haemoglobin > 11 g/dL) were allocated to the reference group.

Exclusion criteria for all subjects were as follows: amenorrhea (lack of menstruation in the 3 months prior to the study), menopause, iron-metabolism-related diseases, chronic gastric diseases, renal disease, or blood donor status. For the nutritional intervention groups, an additional exclusion criterion was allergy to any of the dairy components used in the assay.

The variable used to calculate the sample size of the nutritional intervention groups was ferritin. Sample size was based on 90% power to detect a minimum difference of 10 units of ferritin between groups with a confidence level of 95%, using a reference mean value of 26 ± 16 ng/mL, previously obtained by our research group in iron-deficient women. It was determined that a minimum of 54 subjects was required in each group.

A total of 584 women contacted the research group to receive information, and 289 underwent the screening process. Women who did not meet the inclusion criteria or declined to participate were excluded (*n* = 104). Finally, a total of 129 iron-deficient women agreed to participate in the nutritional intervention and were randomly distributed into two groups (D-placebo or D-fortified), while 56 iron-sufficient women were included in the reference group. During the study, 16 women abandoned the nutritional intervention and 4 were excluded due to lack of compliance. The results of the 109 women who completed the nutritional intervention (D-placebo, *n* = 54 and D-fortified, *n* = 55) were analysed.

The present study was conducted according to the guidelines laid down in the Declaration of Helsinki, and all procedures were approved by the Clinical Research Ethics Committee of Hospital Puerta de Hierro, Majadahonda, Madrid, Spain (#266, 23 May 2011). All subjects signed informed consent.

### 2.2. Nutritional Intervention Design

The study was performed in Madrid, Spain (latitude: 40.24°N). The intervention in the iron-deficient women consisted of a 16-week long, randomised, placebo-controlled, double-blind, parallel-design trial. The assay was performed all at once starting all volunteers in January. As part of their usual diet, one group consumed 500 mL/day of a dairy product fortified with iron but without vitamin D (D-placebo, *n* = 54) whereas the other consumed 500 mL/day of the product fortified with iron and vitamin D (D-fortified, *n* = 55). This trial followed the CONSORT guidelines [21], details of the flow chart have been previously reported [14]. The study is registered at clinicaltrials.gov as NCT01739907.

The dairy products were manufactured from vanilla-flavoured skimmed milk and supplied 15 mg of iron per unit, in the form of microencapsulated iron pyrophosphate coated with lecithin, equivalent to the 83.3% of Recommended Dietary Allowance (RDA) [15,16,22] and were provided in 500 mL cartons. In addition, the vitamin D-fortified dairy product provided 200 IU (5 μg) of vitamin D_3_ per carton, equivalent to 100% of the daily Spanish RDA [15,16]. Composition of the products is shown on Table 1 (products and data provided by CAPSA, Granda-Siero, Spain). Vitamin D_3_ content in the vitamin D-fortified product was determined by Mass Spectrometer (LC QQQ 6460, Agilent Technologies, Englewood, CA, USA).

Participants were instructed to consume one 500 mL carton, shaken, in one sitting, every day, at least 2 h before or after a meal. Volunteers who could not drink the dairy product one day were instructed to consume two cartons the following day.

**Table 1:** Composition of the dairy products (per 100 mL). Ingredients: skimmed milk, sugar, sucralose, stabilizers (E-452, E-412, E-407, E-339), carotens (E-160a), vanilla flavouring, microencapsulated ferric pyrophosphate, and cholecalciferol (D-fortified product).

Component	D-Placebo Dairy Product	D-Fortified Dairy Product
Energy (kcal)	37 (156 kJ)	37 (156 kJ)
Proteins (g)	3.1	3.1
Carbohydrates (g)	5.4	5.4
Fat (g)	0.3	0.3
Of which saturated (g)	0.2	0.2
Calcium (mg)	120	120
Phosphorous (mg)	97	97
Iron (mg)	3.1	2.9
Vitamin D (µg)	0	0.99

### 2.3. Dietary Control, Health Questionnaires, and Anthropometric Determinations

Dietary intake was evaluated at baseline; subjects completed a 72 h detailed dietary intake report specifying the type and/or brand of the product and serving weights of food consumed. Daily food, energy and nutrient intake, and energy provided by macronutrients were calculated by a computer application (DIAL, Alce Ingeniería, Madrid, Spain).

Participants of the intervention groups were instructed not to deviate from their regular lifestyle and to maintain their normal diet and exercise habits. In addition, volunteers completed questionnaires every month in order to monitor possible health problems, menstruation changes, medication use (including hormonal contraceptives and type), diet changes, and changes in their normal routine, including holidays, extra sun exposure or use of tanning beds. The compliance of the study was assessed monthly by questionnaires and a personal interview when the volunteers underwent blood sampling. The volunteer was excluded of the data analysis if more than 4 cartons were left at the end of the assay.

At baseline and at 8 and 16 weeks, body weight was measured with a scale (to a precision of 100 g; Seca, Hamburg, Germany), height was measured with a stadiometer incorporated into the scale, and body mass index (BMI) was calculated. Systolic and diastolic blood pressure was measured with a validated digital automated blood pressure monitor (OMROM M6; Omrom Health Care Co., Kyoto, Japan). To avoid inter-examiner variability, one trained member of the research team did all anthropometric and blood pressure determinations, always under the same conditions.

### 2.4. Blood Sampling and Biochemical Analyses

This article presents secondary outcomes of a wider study. Iron and bone determinations were reported previously [14,23]. Blood samples were collected at baseline and at weeks 8 and 16 after a 12 h fasting period. Haemoglobin was determined in whole blood following standard laboratory techniques using the Symex NE 9100 automated haematology analyser (Symex, Kobe, Japan). Serum ferritin was determined by the Modular Analytics Serum Work Area analyser (Roche, Basel, Switzerland). Serum 25OHD was analysed by an ELISA commercial kit (25-hydroxyvitamin D EIA, Immunodiagnostic Systems, IDS, Boldon Colliery, UK), with intra- and inter-assay coefficients of variation of 5.6% and 6.4%, respectively. All determinations of each person were carried out in the same run to avoid interassay variation. Serum total-cholesterol (T-chol), HDL-cholesterol (HDL-chol), LDL-cholesterol (LDL-chol), triglycerides and glucose were determined by an automatic analyser (RA 2000; Technicon, Tarrytown, NY, USA). The cardiovascular risk index was calculated as the T-chol/HDL-chol ratio.

All determinations were subject to the ISO-9001-2000 requirements.

### 2.5. Statistical Analyses

Data are presented as means with their standard deviations. A normal distribution of variables was determined by the Kolmogorov-Smirnov test. Triglyceride values were log-transformed for statistical testing. Pearson’s linear correlation tests between parameters were performed at baseline for all volunteers (*n* = 165). Repeated measures analysis of variance (ANOVA) was carried out for time and time × group effects for the nutritional intervention groups (*n* = 109). When a significant effect was observed, the post hoc Bonferroni test was used. A *p* value of <0.05 was considered significant. The SPSS statistical package for Windows (version 20.0, IBM, Armonk, NY, USA) was used to analyse the data.

## 3. Results

A total of 165 women completed the study with a compliance rate >96%. Ages of the volunteers were 24.7 ± 4.3 and 26.5 ± 3.8 years for the intervention and reference groups, respectively, without significant differences between groups. Mean baseline haemoglobin value was 13.4 ± 0.8 mg/dL for all women, without significant differences between groups. Ferritin levels were 72.1 ± 29.5 ng/mL for the reference group and 24.7 ± 12.5 ng/dL for the intervention groups (*p* < 0.001). No differences were found in energy, macronutrient intake, or dietary lipid profile at baseline (Table 2).

The reference group did not show significant differences compared to the nutritional intervention groups at baseline in 25OHD (51.7 ± 17.8 nmol/L) or the rest of parameters. Serum 25OHD and T-chol (*r* = −0.176, *p* = 0.023), and serum 25OHD and LDL-chol (*r* = −0.176, *p* = 0.024) were inversely correlated. No other significant correlations were found (*n* = 165).

Body weight presented significant time effects in the D-placebo and D-fortified groups (*p* < 0.001 and *p* = 0.003 respectively), with higher values at weeks 8 and 16 compared to baseline (Table 3). Body mass index was within normal limits throughout the study in all volunteers (<25 kg/m^2^).

**Table 2:** Baseline energy, nutrient and cholesterol intakes of the three groups.

**Component**	**Experimental groups**		
	**D-placebo (*n* = 54)**	**D-fortified (*n* = 55)**	**Reference (*n* = 56)**
Energy (kcal/day)	2057 ± 515	2095 ± 528	2122 ± 487
Protein (% energy/day)	16.9 ± 3.5	16.3 ± 3.7	15.4 ± 3.1
Carbohydrate (% energy/day)	40.3 ± 6.4	38.7 ± 6.4	40.9 ± 6.7
Lipid (% energy/day)	39.4 ± 6.4	41.4 ± 5.8	40.0 ± 6.7
SFA (% energy/day)	13.2 ± 3.1	14.0 ± 3.2	13.0 ± 3.0
MUFA (% energy/day)	17.0 ± 3.5	17.4 ± 3.4	17.4 ± 4.0
PUFA (% energy/day)	5.4 ± 1.4	5.5 ± 1.8	5.4 ± 1.8
Cholesterol (mg/day)	316.8 ± 131.4	344.1 ± 190.3	305.5 ± 113.3
Calcium (mg)	910 ± 319	945 ± 362	909 ± 313
Vitamin D (μg)	3.2 ± 2.4	2.8 ± 3.1	4.0 ± 5.0
Iron (mg)	13.5 ± 5.3	12.9 ± 5.2	13.0 ± 5.0

D-placebo, women assigned to the iron-fortified skimmed milk; D-fortified, women assigned to the iron and vitamin D-fortified skimmed milk. SFA, saturated fatty acids; MUFA, monounsaturated fatty acids; PUFA, polyunsaturated fatty acids. Values are means ± SD. No differences were found between groups (ANOVA).

**Table 3:** Body weight, glucose, lipids and blood pressure of the intervention groups.

Groups	Baseline	Week 8	Week 16	Time effect (*p*)
*Body Weight (kg)*				
D-placebo	59.9 ± 8.3	60.5 ± 8.6 *	60.8 ± 9.0 *	<0.001
D-fortified	59.1 ± 9.4	59.7 ± 9.1 *	59.8 ± 9.1 *	0.003
*Serum 25OHD (nmol/L)*				
D-placebo	62.9 ± 20.8	59.4 ± 19.6*	63.2 ± 18.3	0.010
D-fortified	62.3 ± 20.8	67.1 ± 19.8*	71.2 ± 21.1 *^,#^	<0.001
*Glucose (mg/dL)*				
D-placebo	83.40 ± 5.85	83.25 ± 5.42	83.68 ± 5.08	NS
D-fortified	83.77 ± 6.32	84.02 ± 6.28	83.14 ± 6.34	NS
*T-Chol (mg/dL)*				
D-placebo	176.81 ± 34.04	179.53 ± 35.66	182.64 ± 37.21	NS
D-fortified	176.51 ± 40.64	177.98 ± 39.93	175.57 ± 29.73	NS
*LDL-Chol (mg/dL)*				
D-placebo	103.27 ± 29.61	101.34 ± 29.43	107.36 ± 32.80 *^,#^	0.005
D-fortified	101.16 ± 29.32	99.09 ± 28.15	96.95 ± 26.82	NS (0.07)
*HDL-Chol (mg/dL)*				
D-placebo	66.40 ± 14.04	66.25 ± 12.71	66.42 ± 11.97	NS
D-fortified	69.79 ± 14.23	69.51 ± 15.12	69.30 ± 11.91	NS
*Triglyceride (mg/dL)*				
D-placebo	72.04 ± 29.65	76.68 ± 30.26	75.13 ± 26.33	NS
D-fortified	74.06 ± 31.92	79.41 ± 41.44	77.86 ± 32.95	NS
*T-Chol/HDL-Chol*				
D-placebo	2.73 ± 0.54	2.78 ± 0.62	2.80 ± 0.63	NS
D-fortified	2.54 ± 0.55	2.59 ± 0.63	2.59 ± 0.55	NS
*Systolic BP(mmHg)*				
D-placebo	107.7 ± 11.7	108.4 ± 9.9	108.3 ± 9.4	NS
D-fortified	109.3 ± 10.4	108.6 ± 8.2	105.9 ± 9.1 ^#^	0.017
*Diastolic BP (mmHg)*				
D-placebo	67.1 ± 8.3	66.3 ± 6.9	66.7 ± 7.5	NS
D-fortified	69.2 ± 9.4	66.0 ± 7.0 *	66.6 ± 7.3 *	0.010

D-placebo, women assigned to the iron-fortified skimmed milk; D-fortified, women assigned to the iron and vitamin D-fortified skimmed milk; BP, blood pressure. Values are means ± SD. Time × group interaction was not significant except for LDL-chol (*p* = 0.001). * *p*< 0.05 compared to baseline; ^#^ *p* < 0.05 compared to week 8.

Serum 25OHD levels increased from baseline to week 16 in the D-fortified group but not in the D-placebo group which showed a decrease at week 8. Serum glucose and lipid levels were within normal limits, with no differences between groups at baseline (Table 3). No changes over time or group were found for glucose or lipids, except for LDL-chol (time × group, *p* = 0.001), which increased in the D-placebo group (*p* = 0.005) but tended to decrease in the D-fortified group (*p* = 0.07) without significant differences between groups at week 16 (*p* = 0.07). Systolic and diastolic blood pressure did not present time × group interactions; both values decreased significantly (*p* = 0.017 and *p* = 0.010, respectively) in the D-fortified group whereas in the D-placebo group no changes were observed (Table 3).

## 4. Discussion

This study was carried out in healthy women whose lipid and glucose levels and blood pressure were within normal limits. The nutritional intervention was performed in women with low iron stores who, on average, presented vitamin D insufficiency (25OHD levels between 51 and 74 nmol/L), and those who consumed the vitamin D-fortified product nearly reached sufficiency (25OHD, 71 ± 21 nmol/L) at the end of the study. However, although both dairy products contained added iron, we previously demonstrated that the iron salt used to fortify the food (microencapsulated ferric pyrophosphate) was not effective at improving iron status, due to poor absorption [14]. Therefore, present results are attributed to vitamin D.

The slight increase in body weight seen in both intervention groups indicates high compliance, as both skimmed milks added an additional 185 kcal per day to the habitual diet that did not change throughout the study [14]. Nevertheless, an adaptation to the extra calories may occur, as body weight remained stable from week 8 to the end of the study and none of the women were or became overweight.

Some studies suggest a potential benefit of vitamin D in glucose homeostasis, most of them obtained in observational studies and in type 2 diabetes patients [10,24]. A recent systematic review [25] concluded that there is a weak effect of vitamin D supplementation in reducing fasting glucose and improving insulin resistance in patients with type 2 diabetes or impaired glucose tolerance, but not in subjects with normal glucose tolerance. This is in agreement with our findings as the volunteers were healthy young women with glucose and lipid parameters within normal limits.

The negative association between 25OHD and T-chol and LDL-chol values found in the present study concurs with several reports in different population groups [26,27]. During the intervention a non-significant decrease in LDL-chol was observed in the D-fortified group while in the D-placebo group it increased. These changes may be attributed to the hypercholesterolaemic effect of milk casein [28,29], as the dairy product was skimmed, and to a counteracting effect of vitamin D. In this regard, there were no confounding dietary factors; the consumption of the dairy products increased the percentage of energy from proteins and reduced that from fat in both groups; and there were no differences in macro and micronutrient intake, other than vitamin D, between groups [14]. Some studies, however, reported a positive relationship between vitamin D status and serum lipids [7,30,31], and Ponda *et al.* [32] observed that correcting vitamin D deficiency by administering 50,000 IU of vitamin D weekly during 8 weeks significantly increased LDL-cholesterol in vitamin D-deficient high cardiovascular-risk adults. The discrepancy between this finding and ours can be interpreted considering the much higher dose given, compared to 200 IU/day in the present study, and suggests that the relationship between the vitamin D dose and LDL-cholesterol levels follows a U trend.

To the best of our knowledge, no other randomised controlled trial with a vitamin D-fortified food has observed a significant decrease in blood pressure. It has been suggested that components of milk such as calcium, potassium, magnesium, casein, and whey protein, protect against hypertension [33], and Wang *et al.* [34] found that intakes of low-fat dairy products, calcium, and vitamin D were each inversely associated with risk of hypertension in a cohort of women (age ≥ 45 years). Similarly, in another prospective study conducted in Spain, low-fat (but not whole-fat) dairy product consumption was associated with a lower risk of hypertension [35]. The possible effect of calcium and milk proteins on blood pressure was not observed in the present study, as blood pressure values did not vary in the D-placebo group. One explanation for this is the fact that calcium intake was adequate and reached 100% of the Spanish RDA (900 mg/day) at baseline [15,16]. Additionally, the quantity of milk proteins provided by these dairy products was lower than in other studies. Researchers who found a beneficial effect of whey and casein supplementation on blood pressure administered 40–50 g/day of protein [36,37] while in our intervention the extra consumption of milk protein was of only 15 g/day.

Therefore, we attribute the beneficial effects of this iron and vitamin D-fortified dairy product on blood pressure to vitamin D. Increasing evidence associates this vitamin with the renin-angiotensin-aldosterone system, as the vitamin D-receptor is expressed in the juxtaglomerular apparatus and its activation decreases renin gene expression [38]. Along these same lines, in a cohort of coronary syndrome patients, Tomaschitz *et al.* [39] found that serum 25OHD and 1,25-dihydroxyvitamin D levels were related to lower plasma renin concentrations and angiotensin 2 levels. Another mechanism by which vitamin D may affect blood pressure is secondary to parathormone changes [38], however parathormone levels in the present intervention were stable [23]. Consequently, the results of this randomised placebo-controlled trial show that consumption of a daily physiological dose of cholecalciferol (*i.e.*, the Spanish RDA) in a fortified food was able to significantly decrease blood pressure, and support a direct effect of vitamin D metabolites on the renin-angiotensin-aldosterone system and blood pressure.

There is controversy on the dietary recommendations on vitamin D. For the United States of America and Canada, the Institute of Medicine has established in 2010 a RDA of 600 IU (15 μg) per day for adults and an Estimated Average Requirement of 400 IU (10 μg) per day [17]. In Spain, RDA is currently set at 200 IU (5 μg) per day [15,16], however European reference values are under revision. Present data show that mean vitamin D intake was lower than 5 μg (Table 2) and that the consumption of the D-fortified dairy product improved vitamin D status. The amount of vitamin D added to the product was in line with the recent European Food Safety Authority report regarding claims of vitamin D on bone health maintenance [40]. Therefore, the promotion of this type of product may have beneficial health effects, as the Spanish population is not aware of the need for extra intake of this vitamin, even in the winter season.

It is notable that this slight but significant result is observed in healthy women. According to the Framingham Heart Study [41], the Adult Treatment Panel-III (ATP-III) [42], and the European Society of Cardiology [43] score charts, our volunteers were at very low coronary heart disease risk, as determined by a combination of the following parameters: blood pressure <120/<80 mmHg, T-chol <199 mg/dL and HDL-chol ≥55 mg/dL, in addition to being non-smokers and non-diabetics.

Factors that could have affected the results were monitored. Thirty percent of the participants were using hormonal contraceptives during the study. Moller *et al.* [44] found, in a relative small sample of Danish women, that hormonal contraception was associated with higher concentrations of vitamin D metabolites. However, in the present study differences in 25OHD between users and non-users of hormonal contraceptives were not significant (data not shown), and participants did not change their use throughout the study; therefore this cannot be seen as a confounding factor.

The limitations of the study are the fact that women were healthy and all parameters were within normal limits, that only one level of fortification was studied and that the effects of fortified food was not compared to that of supplements. Further studies regarding the effects of pharmacological *versus* nutritional doses of vitamin D on cardiovascular risk are needed. Moreover, investigations should be performed on the metabolic effects of the consumption of vitamin D-fortified foods by vitamin D-deficient individuals, as well as by hypertensive, cardiovascular, and metabolic syndrome patients.

## 5. Conclusions

In conclusion, 25OHD is negatively associated with T-chol and LDL-chol in young menstruating women. Consumption of a dairy product fortified with vitamin D (5 μg, 200 IU per day) did not change lipid levels, however, reduced slightly—but significantly—systolic and diastolic blood pressure in normotensive women with low iron stores.
